# Sputum smear microscopy in DOTS: Are three samples necessary? An analysis and its implications in tuberculosis control

**DOI:** 10.4103/0970-2113.45196

**Published:** 2009

**Authors:** Sukhesh Rao

**Affiliations:** *Department of Tuberculosis and Respiratory Diseases, Yenepoya Medical College, Deralakatte, Mangalore - 575 018, Karnataka, India*

**Keywords:** Acid fast bacilli, DOTS, SMS

## Abstract

**Objectives:**

To assess relevance of spot morning spot (SMS) method of smear microscopy in tuberculosis control by directly observed treatment short course (DOTS).

**Materials and Methods:**

Screening of smear microscopy results of 546 cases of pulmonary tuberculosis at our DOTS centre.

**Results:**

Among 546 cases, 82% had all three samples positive for AFB, 98% had either morning sample or spot and morning sample positive. 2% had second spot sample positive.

**Conclusion:**

Examination of third sample, especially second spot sample, does not add significantly to the diagnostic yield. Examination of one spot and early morning samples were able to correctly diagnose 98% cases. This has strong implications in DOTS strategies.

## INTRODUCTION

In the diagnosis of tuberculosis, detection of acid fast bacilli (AFB) on microscopic examination of sputum smears remains the most widely used investigation in clinical practice, especially in developing countries and countries with high prevalence.^1^,^2^

As this also differentiates the infectious from the noninfectious cases, sputum smear microscopy represents one of the five pillars in the directly observed treatment short course (DOTS) strategy for tuberculosis control.^3^ Unfortunately, this has its pitfalls because the positivity depends on the number of bacilli, type of lesions, etc.^4^ Some studies have shown that the diagnostic yield increases with each specimen that is examined^5^,^6^ leading to the recommendation that serial sputum smear examination is better than single sample. WHO in its tuberculosis control program strategy recommends spot, morning, spot (SMS) sampling method. This study was undertaken to assess whether this is cost- beneficial or is there any incremental benefit by increasing the number of samples.

## MATERIALS AND METHODS

Since 2004, all patients of tuberculosis visiting our center are diagnosed and treated as per DOTS protocol. A retrospective study of cases of pulmonary tuberculosis was undertaken. Records of such patients were screened and analyzed with regards to their sputum smear positivity. All the three samples were taken into account. No other details or findings were noted or evaluated.

## RESULTS

Records of 546 cases were screened. Of these, 447 (82%) patients had all their three sputum samples positive for AFB. When analyzing the samples separately it was found that 32 (6%) patients had only their early morning sample positive, while another 9% (49) had two of the three samples positive. A very small number, that is, 12 (2%) patients had their second spot sputum sample positive, thereby implying that this number was picked only on the examination of the third sample.

## DISCUSSION

Several studies have shown that three serial sputum smear examination is ideal for diagnosis of pulmonary tuberculosis cases. The samples may vary from three consecutive early morning samples or SMS samples as a part of DOTS strategy. However, there are also some conflicting reports on this.^7^,^8^ Anecdotal evidence also led to this observation. Results of this study also confirm this.

As it is very clear that 447 (82%) out of 546 patients had entire three samples positive for AFB. After various permutations and combinations, [Table 1] it was found that almost 98% cases would have been diagnosed by analyzing only two samples (spot and early morning). The examination of the third sample added only 2% to the diagnostic yield. Though 2% increase in yield in infectious diseases like tuberculosis cannot be neglected, yet it is worthwhile to ponder whether the third sample examination should be included as a program strategy or limited to a select group of patients.

**Table 1:** Sputum smear details with reference to the tested sample

Analyzed group	Sputum samples (S_1_ MS_2_*)	Number	Percentage
All three samples positive	S_1_ MS_2_ (+ve)	447	82
Two of the samples positive	a) S_1_ −ve, MS_2_ +ve	15	2.7
	b) M −ve, S_1_S_2_ +ve	10	2
	c) S_2_ −ve, S_1_M +ve	24	4.3
One of the sample positive	a) S_1_M −ve, S_2_ +ve	12	2
	b) S_1_S_2_ −ve, M +ve	32	6
	c) MS_2_ −ve, S_1_ +ve	6	1

* S_1_= First spot sample; M= Early morning sample; S_2_= Second spot sample

Moreover, if we take total samples into consideration it is clear that almost 10% had single sputum sample positive which would have necessitated a chest skiagram for diagnosis. Though this is significant, yet we feel that overall beneficial effect of reducing the second spot sample would be substantial.

Besides these two features, this study has the limitation of being a retrospective study due to which the correlation with clinical features, duration, and extent of disease with the sputum status could not be analyzed which would definitely have added to our interpretation. A study to include these data has been initiated and we hope results will definitely help us to evolve better strategies in future. Until such studies throw more light on the findings, no significant revision in quality assurance protocol of the program is recommended. Nevertheless, we feel that in the light of the present observations, the examination of the third sample as a routine policy in DOTS program need to be reviewed in the background of cost, time, and manpower involved and the advantages obtained by it. This becomes highly significant in countries with high prevalence and poor resources.
